# Supplementary material for: The composition and structure of the ubiquitous hydrocarbon contamination on van der Waals materials
Source: Nat Commun. 2022 Nov 9;13:6770. doi: 10.1038/s41467-022-34641-7 (PMC9646725; doi:10.1038/s41467-022-34641-7)
Supplement: Supplementary file 1 — supplementary information [file 41467_2022_34641_MOESM1_ESM.pdf]

Supplementary Information for:

The composition and structure of the ubiquitous hydrocarbon contamination on van der Waals materials

András Pálinkás[1*], György Kálvin[1], Péter Vancsó[1], Konrád Kandrai[1], Márton Szendrő[1], Gergely Németh[2], Miklós Németh[3], Áron Pekker[2], József S. Pap[3], Péter Petrik[1,4], Katalin Kamarás[2], Levente Tapasztó[1] and Péter Nemes-Incze[1*]

[1]Centre for Energy Research, Institute of Technical Physics and Materials Science, 1121 Budapest, Hungary

[2]Wigner Research Centre for Physics, Institute for Solid State Physics and Optics, 1121 Budapest, Hungary

[3]Centre for Energy Research, Institute for Energy Security and Environmental Safety, 1121 Budapest, Hungary

[4]University of Debrecen, Department of Electrical and Electronic Engineering, 4032 Debrecen, Hungary

[*]corresponding authors: andras.palinkas@ek-cer.hu, nemes.incze.peter@ek-cer.hu

Supplementary Figure 1. – other, volatile contaminations

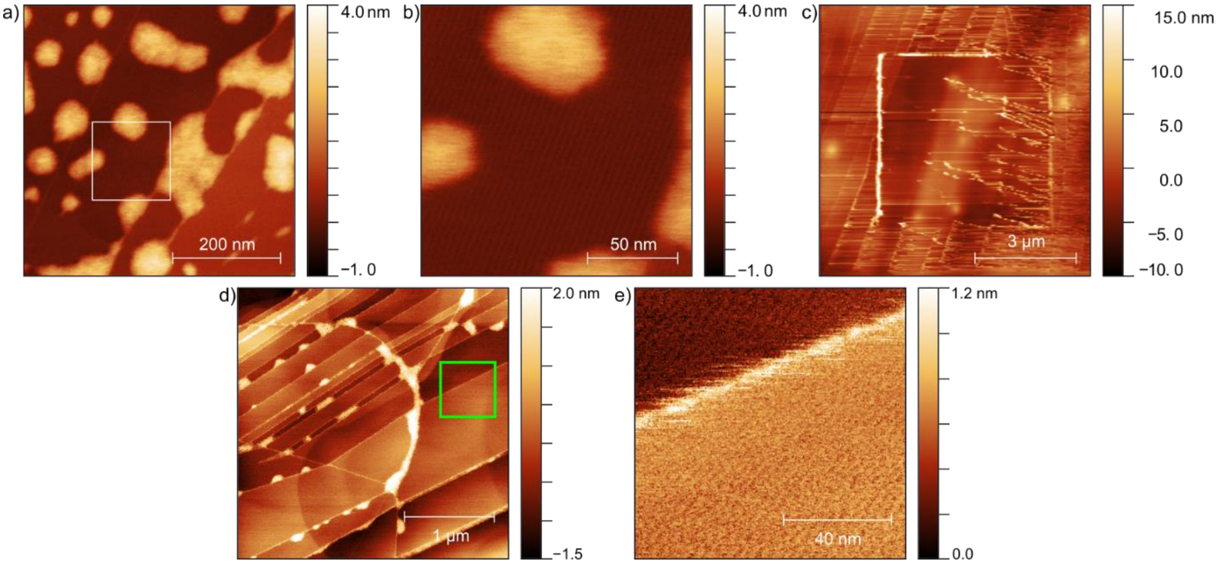

Supplementary Fig. 1. a) 500×500 nm PF AFM image on HOPG after aging in ambient laboratory conditions. Liquid droplets condensed on to the graphite surface. b) Higher magnification PF AFM image in the position marked with white rectangle on a). The stripe structure of the airborne alkane layer could be observed on the atomically flat terraces, not covered by droplets. c) The liquid droplets can be swept out in contact mode scanning even with low normal forces. d) After an hour-long exposure to 10^{-2} Torr vacuum significantly fewer droplets can be seen on the surface. e) The stripe pattern can maintain its periodicity and direction through atomic steps.

As it can be observed in Supplementary Fig. 1a, in many cases liquid droplets also condensed onto the flat surface of the vdW materials after a few days storage. We note that the droplets are not always present, and they are more likely to form on bulk HOPG samples than on exfoliated graphite, graphene or hBN. Nevertheless, the stripes can be imaged between the droplets (Supplementary Fig. 1b) or, if they swept out by a scanning AFM tip in contact mode (Supplementary Fig. 1c). Although not strictly necessary, we found that an hour-long exposure to a 10^{-2} Torr vacuum, makes the imaging of the stripes easier, possibly because the other volatile contaminations (e.g. water) are taken away (Supplementary Fig. 1d), while the adsorbate layer was stable, i.e. did not desorb from the surface (see Supplementary Fig. 1). The airborne alkane layer itself was stable under UHV (5×10^{-11} Torr) for weeks.

Supplementary Figure 2. – Graphene bubble

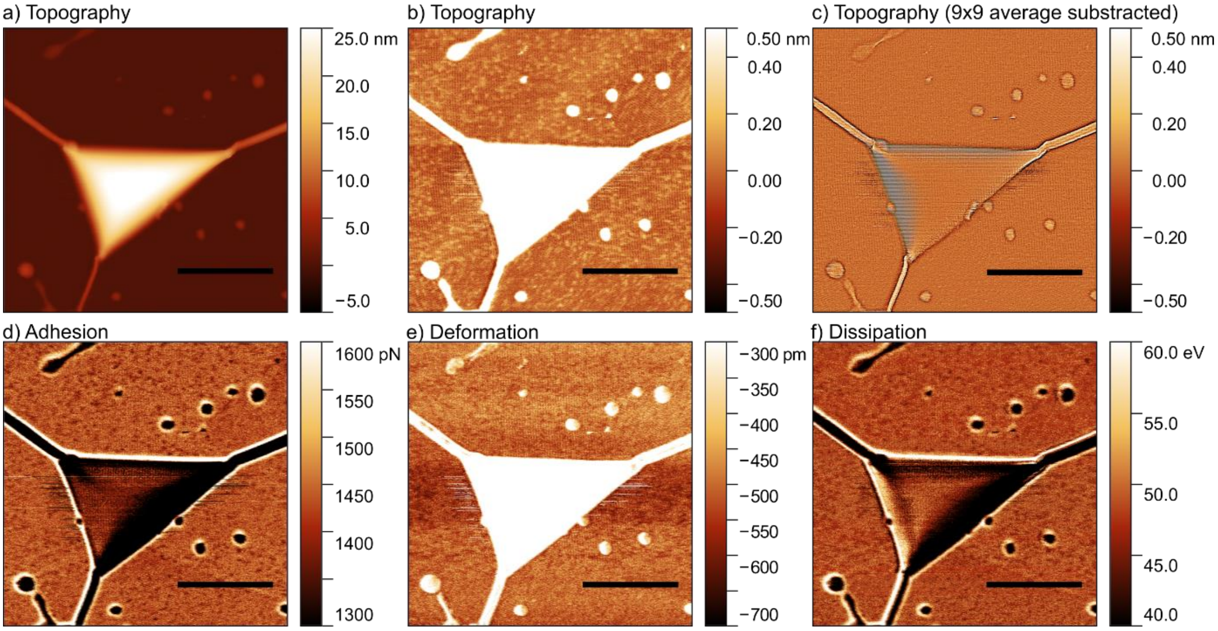

Supplementary Fig. 2. 500×500 nm PF AFM image of a triangular graphene bubble in a graphene/hBN heterostructure. The stripe pattern can be observed not only on the Topography channel, but also in Adhesion, Deformation or Dissipation. The c) image calculated using only the Topography channel, 9×9 average filter subtracted, as a background to visualize the stripe pattern even on the crest of the bubble. (Scale bars: 200 nm)

We found that the stripes can climb up to bubbles which formed on the hBN/graphene interface. The maximum height of the triangular graphene bubble in Supplementary Fig. 2a is 28 nm respect to the hBN substrate is two magnitude larger than the apparent height of the molecular stripes (~0.1-0.2 nm), which results that they remain hidden if we plot the height channel with full scale bar. On the other hand, the stripe pattern is readily visible in the adhesion, deformation and dissipation channels (see Supplementary Fig. 2d-f), showing that they maintain their direction and periodicity (within our experimental error, 5%). By changing the data scale the domain structure of stripe pattern can be brought forward even in the topography channel, on the smooth hBN/graphene areas (Supplementary Fig. 2b). We subtracted a 9×9 average filter background from the topography data to bring forward the stripe structure even on the crest of the bubble.

Universally in vdW heterostructures, there is a 1-2% strain in the monolayer vdW membrane which forms the bubble[1]. Within our experimental error we do not see any difference in the periodicity or in the structure of the stripe pattern when formed on strained (bubble) and unstrained (supported) graphene.

Supplementary Figure 3. – hBN/graphene heterostructure – graphene edge

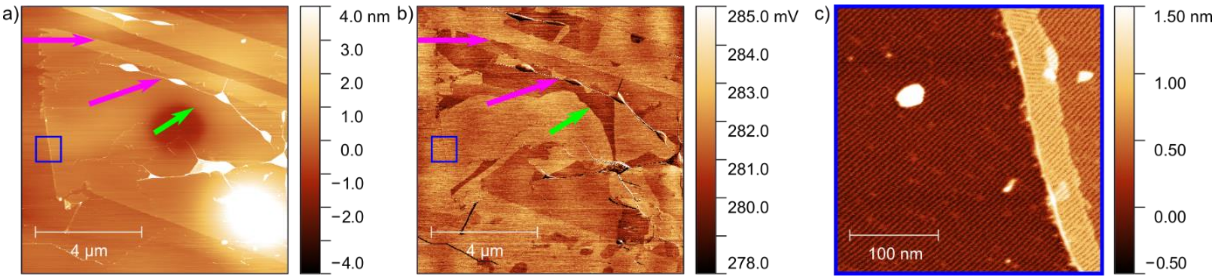

Supplementary Fig. 3. 10×10 µm contact mode AFM image of a ~0° graphene/hBN heterostructure. a) On the Topography channel the edge of the graphene layer and graphene bubbles can be observed. b) On the Torsion signal, a manifold frictional domain pattern can be seen. The borders between the frictional domains occasionally follow the topographical features (e.g. graphene edge, wrinkle or bubbles, purple arrows) and change direction abruptly on atomically flat areas (green arrow). c) The stripe pattern can maintain periodicity and direction through the graphene edge to the hBN substrate. Domains with diameter of few tens of nanometers are also observed.

Supplementary Figure 4. – STM topographic contrast of the molecules at different bias voltages

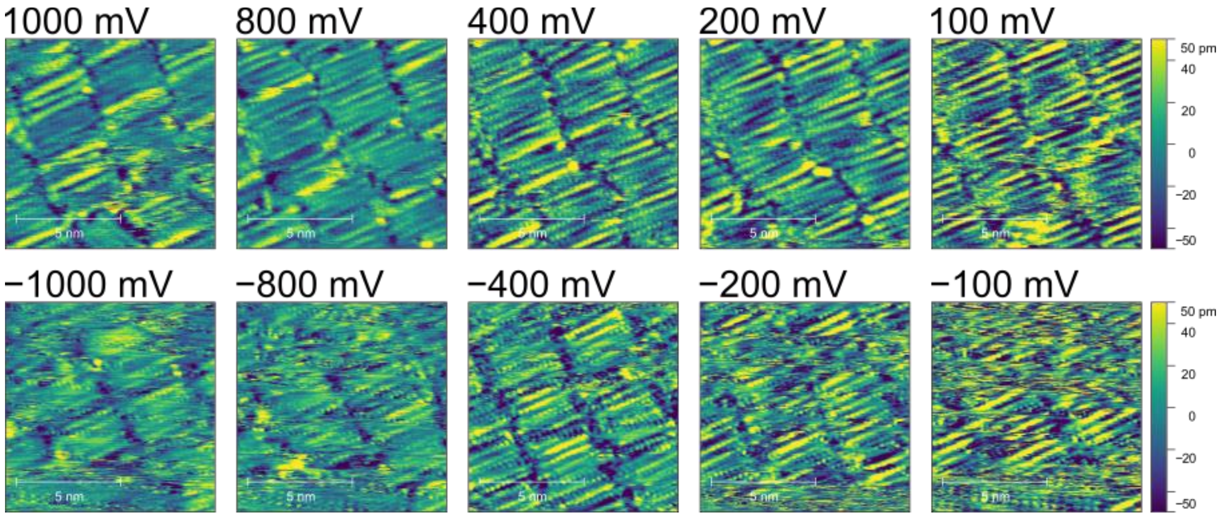

Supplementary Fig. 4. 10×10 nm STM topography images of the airborne molecule layer at different biases. Please note that the images with negative bias values represent a different area.

We did not observe significant contrast change in the STM image of the molecular layer using different bias voltages. This is in agreement with the STS spectra measured on the contaminated graphite in Supplementary Fig. 2 in the main text, which reveals that there are no molecular states in the energy range of ±1 eV in the LDOS. This is expected for normal alkanes, since their lowest unoccupied and highest occupied molecular orbitals are more than 7 eV[2] away from the graphite Fermi level.

Supplementary Figure 5. – XPS

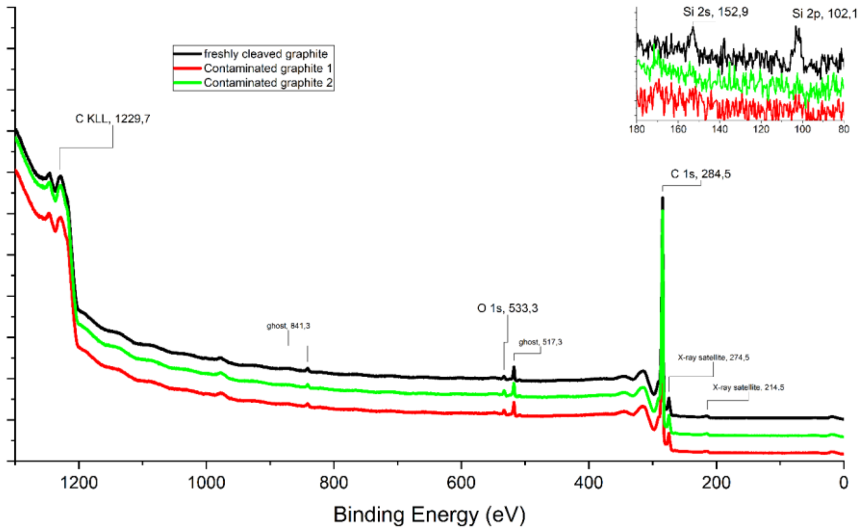

Supplementary Fig. 5. XPS spectra of three HOPG samples. The XPS spectra of the two aged graphite samples (red, green) covered with contaminated molecules, with AFM-confirmed stripe pattern did not show measurable difference from the reference freshly cleaved HOPG substrate. Inset: However, in the reference spectrum low amount of Si (~0.1%) was observed, it did not show up in the spectra of the aged samples.

We have applied XPS to identify the elemental composition of the molecule adlayer, but we could not find measurable difference between the aged (covered with contaminant molecules) and freshly cleaved HOPG samples. The XPS survey spectra shows the core level and Auger lines of the elements on all three samples. This finding suggests that the adlayer is composed of mainly carbon and hydrogen atoms, XPS is essentially insensitive to the latter. We did not detect any other ubiquitous elements, such as Cl (~199eV), Na (~1071 eV), N (~400 eV) and S (~162eV) up to the detection limit of the XPS, which is in the range of ~0.1-0.2%. Oxygen was the only notable exception with a concentration below 1% (see Supplementary Table 1). This was found on the samples regardless of their aging and we attribute its presence to the poor background pressure (10^{-8} Torr) of our equipment. Low amount (~0.1%) of Si was observed in the clean HOPG sample. Other than that, there were no observable difference between the samples.

	surface atomic concentrations / %		
	C	O	Si
HOPG reference	99.4	0.5	0.1
contaminated HOPG 1	99.2	0.8	0
contaminated HOPG 2	99.1	0.9	0

Supplementary Table 1.

Note: These are semi-quantitative results: all the elemental composition calculations were performed, assuming that the samples were homogeneous within the XPS detected volume.

Supplementary Figure 6. – Ellipsometry

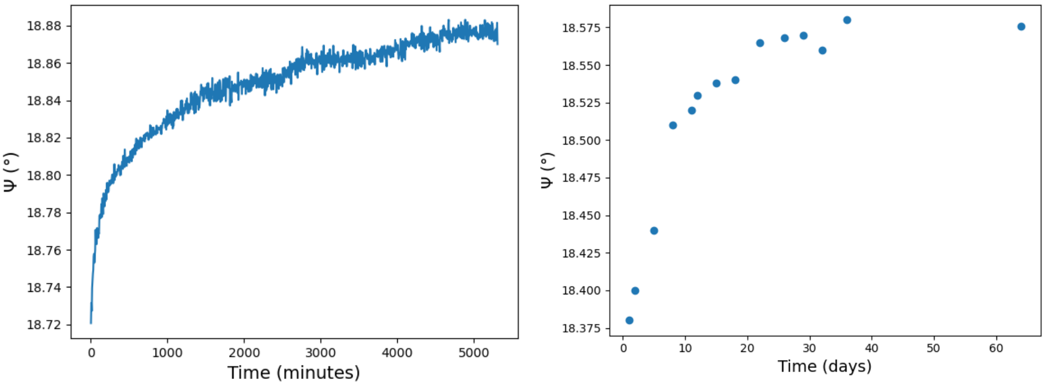

Supplementary Fig. 6. Raw ellipsometry spectra on different time scales measured during adsorption. ($\Psi = \tan^{-1}(|r_p/r_s|)$, where r_p and r_s denote the complex reflection coefficients of light polarized parallel and perpendicular to the plane of incidence, respectively.)

In Supplementary Fig. 6a, one can observe the rapid growing process of an unordered, airborne contamination layer. After the first 40-60 minutes the thickness change of the adsorbate slows down. This first section of contamination growth is described previously for graphite[3–6], hBN[7], MoS_2[8,9] and WS_2[9] layers, and it was shown that the contamination layer is responsible for the hydrophobicity of the surface. We track the process further up to 64 days and find that it is saturated only after 10-14 days.

Supplementary Figure 7. – Friction anisotropy domains caused by $C_{32}H_{66}$ monolayer on graphite

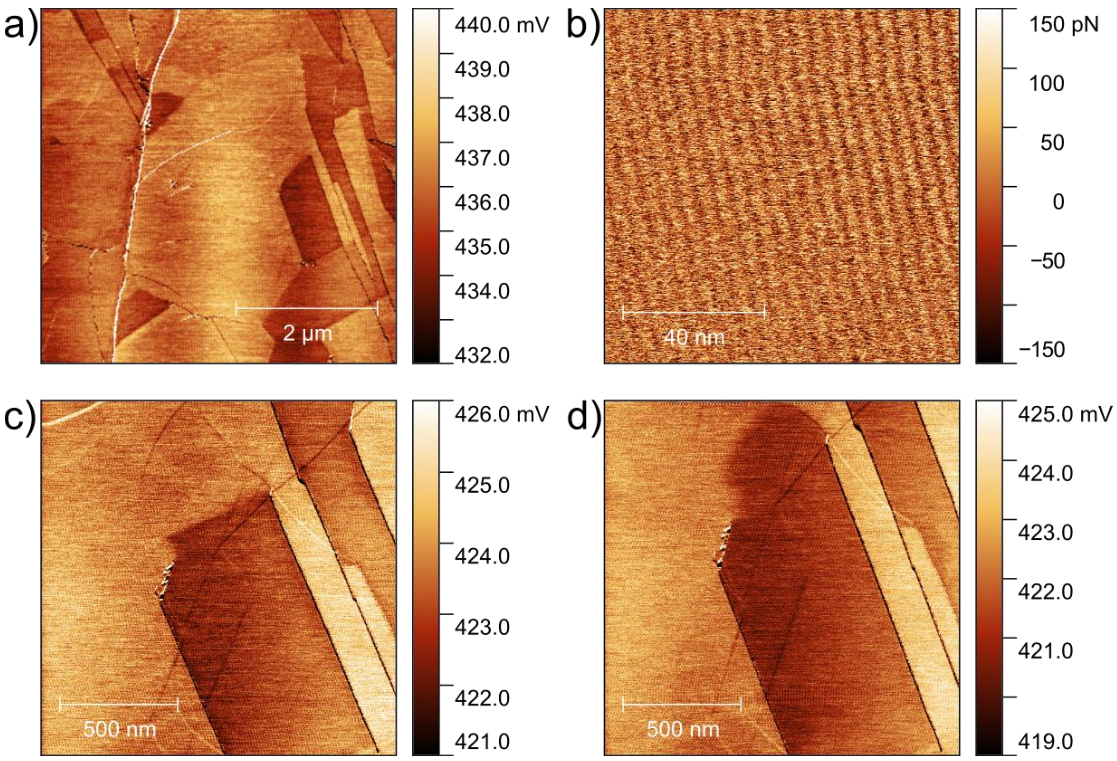

Supplementary Fig. 7. Friction anisotropy domains in the contact mode AFM Torsion signal after covering the freshly cleaved HOPG surface with vapor deposited $C_{32}H_{66}$ monolayer. a) On the larger scale image one can observe the different domains. b) PF AFM Adhesion image of the self-organized monolayer on HOPG. We measured (by AFM) 4.3±0.25 nm for the period of the stripes which is very well agree with the all-trans length (4.22 nm) of $C_{32}H_{66}$. c-d) In two subsequent contact AFM torsion image an abrupt change in the domain structure can be observed.

Supplementary Figure 8. – Extended data to Fig 4 of the main text.

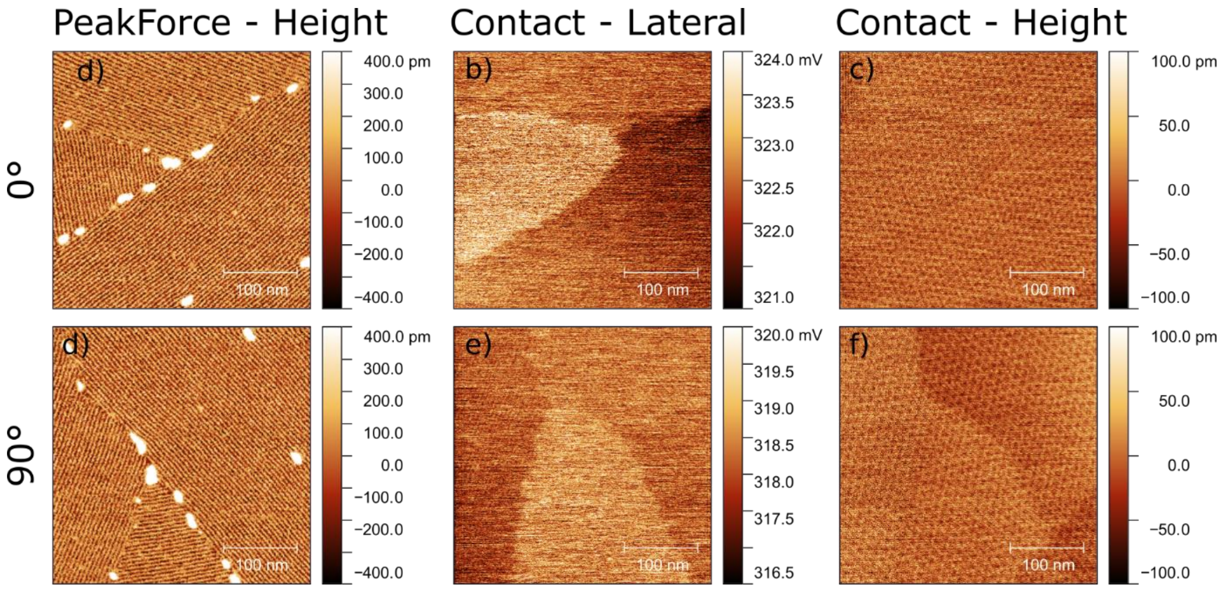

Supplementary Fig. 8. Tripoint boundary in the stripe structure of the contaminant layer on an atomically smooth surface of graphene supported by hBN. a) and d) PF Topography, b) and e) Contact mode torsion, c) and f) Contact mode height channel images in the same area. The upper (a-c) and lower (d-f) images recorded with different fast scanning direction respect to the cantilever's long axis.

In Supplementary Fig. 8 we present extended data to Figure 4 of the main text, from the same 250×250 nm^2 hBN supported graphene area measured with different AFM modes. A tripoint boundary in contaminant layer can be observed in the PF topography maps (a and d), which cannot be seen in contact mode topography (c and f). While there is an intermittent contact between the tip and the sample in every measured pixel in PF mode, the tip is pushed to the sample's surface to a constant deflection (constant force), and it remains in contact during the acquisition of the whole image. In the whole, atomically smooth area an uninterrupted moiré pattern with ~12.4 nm period between the graphene and hBN (<1° rotation) can be observed in the c and f panel. This confirms that (1) there is a perfect contact between the graphene and hBN, and (2) there is no crystal defect, rotation change or strain variation which could be assigned to the domain structure visible in the simultaneously recorded lateral force (friction force) maps in panel b and e.

As one can observe in Supplementary Fig. 8f, there is a crosstalk between the topography and torsion signal in contact mode, which comes from that the horizontal and vertical deflection of the backscattered laser point is not perfectly in line with the four-quadrant photodiode.

It was described earlier[10,11] that the friction anisotropy domains gave a better contrast in the torsion signal if the long axis of the cantilever is parallel to the fast scanning direction (upper panels in Supplementary Fig. 8), than if it is perpendicular (lower panels in Supplementary Fig. 8). For this purpose, we present lateral force maps (recorded with parallel scanning, marked as 0°) in Figure 4 in the main text and elsewhere in the supplementary information, rather than "friction force" maps (perpendicular scanning, marked as 90°).

Supplementary Figure 9. – Heat treatment

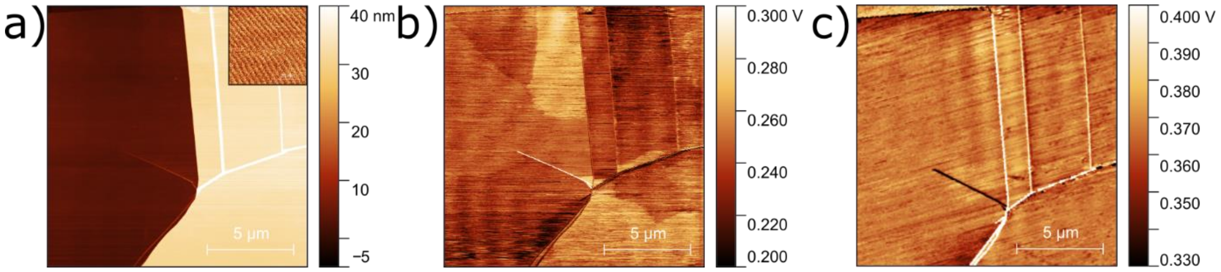

Supplementary Fig. 9. a) Contact topography and b) lateral force images on an exfoliated, naturally contaminated graphite flake. The friction anisotropy domains are visible in the lateral force image. Inset in a) shows the stripe pattern of the adsorbed molecules. c) Lateral force image in the same region after heat treatment at 200 °C. The friction anisotropy domains disappeared, as well as the stripes.

We annealed the samples at 200 °C for 1 h in vacuum at 3×10^{-2} mbar pressure, which resulted in the disappearance of the friction anisotropy domains (see Supplementary Fig. 9) and the stripe structure. Annealing at 200 °C for 1 h under ambient conditions also leads to the disappearance of domains and stripes, but results in less cleaner surfaces. This is due to the desorption of the molecular adlayer, as it was shown that monolayers of normal alkanes with 20-26 carbon atoms desorb from graphitic surfaces between 400 and 500 K[12,13]. The removal of friction domains by annealing is in accordance with the previous findings of Choi et al.[14].

Supplementary Figure 10. – Reshaping of friction anisotropy domains on a thick MoS_2 flake or through the graphene/hBN edge

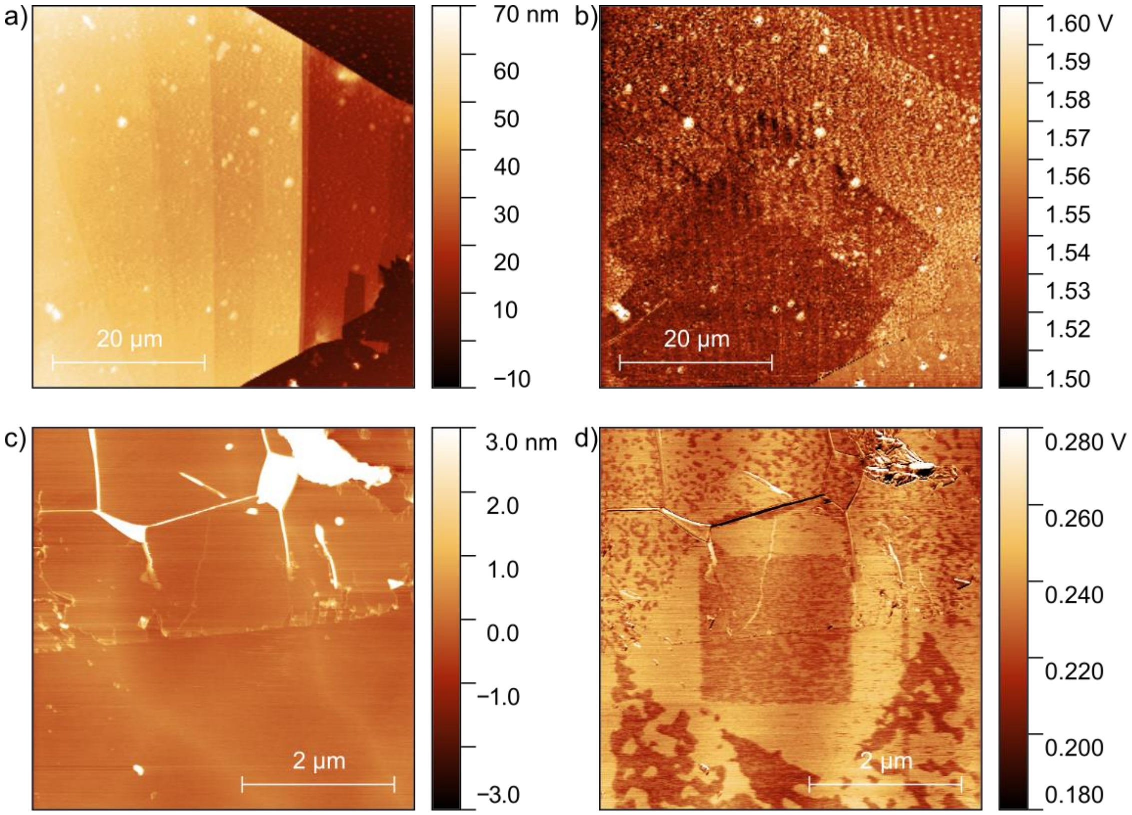

Supplementary Fig. 10. a) Contact topography and b) lateral force images on an exfoliated, naturally contaminated thick MoS_2 flake after reshaping the molecular layer in a 30×30 µm^2 area. c) Contact topography and d) lateral force images in the vicinity of a graphene edge.

We found that the surface of the exfoliated vdW flakes are usually covered not only by the self-ordered alkane monolayer, but by other, unordered airborne particles or condensed vapors as also can be seen in the case of a 50 nm thick, gradual MoS_2 flake in Supplementary Fig. 10a. Despite the simultaneous presence of such unordered particles along the ordered alkane adsorbates, we found that the friction anisotropy domains can be still reshaped by the scanning AFM tip. The formed domain is continuous over the steps between the MoS_2 terraces.

On the other hand, in vdW heterostructures the friction domains can be continuous through chemically and mechanically different layers as it can be seen in Supplementary Fig. 3 in the case of a <1° rotated graphene hBN heterostructure. A similar region is presented in Supplementary Fig. 10c-d where the friction domains were reshaped previously by the scanning AFM tip by the method described in the main text. By using sufficiently high force between the scanning tip and the substrate the domains can be turned to any direction. In the particular case which presented in Supplementary Fig. 10c-d the fast-scanning direction was not set accordingly to the zigzag direction of the substrate thus the deliberately drawn nested friction domains becomes patchy in a few minutes after the drawing process. We explain this phenomenon as the molecules spontaneously rearrange to one of the three preferred zigzag directions of the substrate. Note, that before the spontaneous rearrangement, the formed domains were continuous from the hBN to the graphene covered area, as they do not change orientation related to the graphene's edge, because the graphene is nearly aligned with the hBN substrate (<1° rotation).

Supplementary Figure 11. – Simulations of edge-on and flat-on oriented alkanes in a 1D moiré-cell

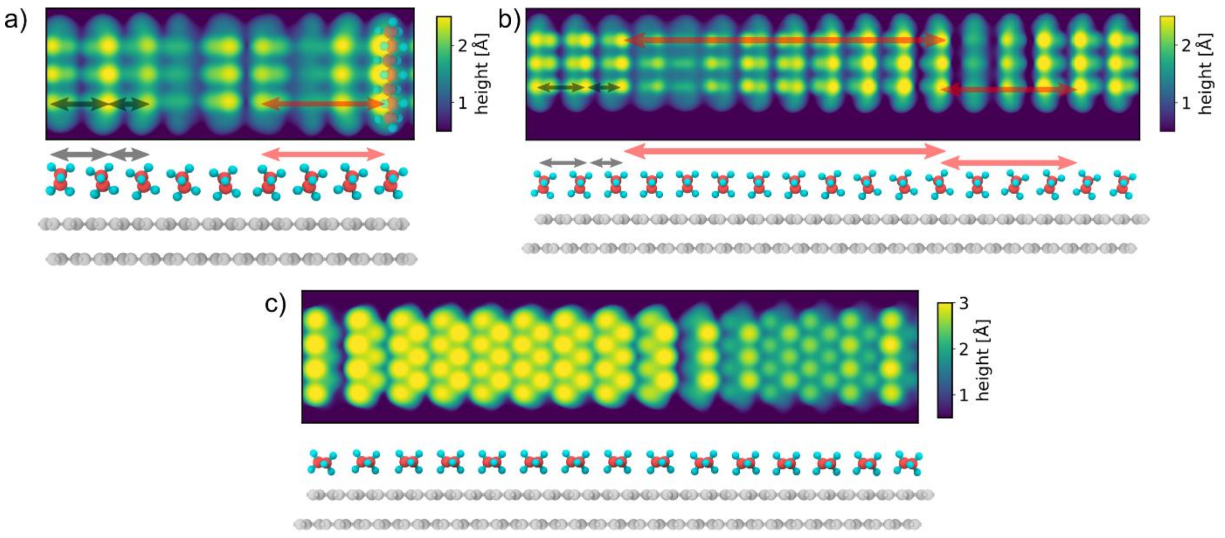

Supplementary Fig. 11. a) Calculated STM image of C_7H_{16} alkanes in the edge-on orientation (same image as in Fig3e of the main text). Alkane – alkane distance is 3.8 Å. b) Calculated STM image of C_7H_{16} alkanes in the edge-on orientation, within the enlarged (4 Å) alkane – alkane distance. c) Calculated STM image of C_7H_{16} alkanes in the flat-on orientation.

In the literature, generally two possible stacking configurations are discussed for alkanes on graphite: the edge-on and flat-on geometries. We have performed DFT calculations in both the edge-on and flat-on orientation of alkanes on graphite (see orientation of the alkane chains on Supplementary Fig. 11). From these two possible stacking configurations the edge-on one is consistent with our STM topography measurements, where the appearance of brighter and darker molecules (quasiperiodic modulation) and the two inter-molecule distances appear (see red and grey arrows in Supplementary Fig. 11), as discussed in the main text. These qualitative signatures of the simulated STM images are demonstrated for both 3.8 Å and 4.0 Å alkane-alkane distances (see Supplementary Fig. 11a and 11b). In the latter case we used 17 alkane chains, where supercell length became 68.2 Å. We also quantified the observed periodicities by using the FFT analysis. We found three peaks (0.378 nm, 0.485 nm and 1.13 nm), where the molecule distance was 3.8 Å and four peaks (0.4 nm, 0.52 nm, 1.7 nm and 3.4 nm) in the case of 4.0 Å distance. The main origins of these periodicities are the same, namely the combined geometrical and electronic structure effects, discussed in detail in the main text. It can be seen that the two edge-on calculations show a tendency in the observed periodicities, where the periodicities in the alkane-graphite system are shifting towards the larger values by increasing the molecular distance. These periodicity values are smaller than the supercell periods of the calculations and could be comparable with the experimentally observed 1D quasiperiodic patterns (of around 1.9 nm period). The main feature of the flat-on orientation is the absence of such complex variability of the observed periodicities. We found only one quasiperiodic modulation, which is equal to the supercell period of the calculation (6.8 nm). This means that inner periodicities do not appear within the supercell in sharp contrast to the edge-on geometry (see Supplementary Fig. 11c). This qualitative difference supports the rather edge-on nature of our findings, because the supercell period of the flat-on geometry is much larger than the observed periodicities in the measurements. Still, it is possible that under various circumstances (temperature, alkane vapor pressure, etc.) the flat-on orientation or mixed phases can be preferred. These mixed phases are beyond our DFT computational limit.

Supplementary Figure 12. – Low temperature structure of the contaminant layer and the C32 layer on HOPG

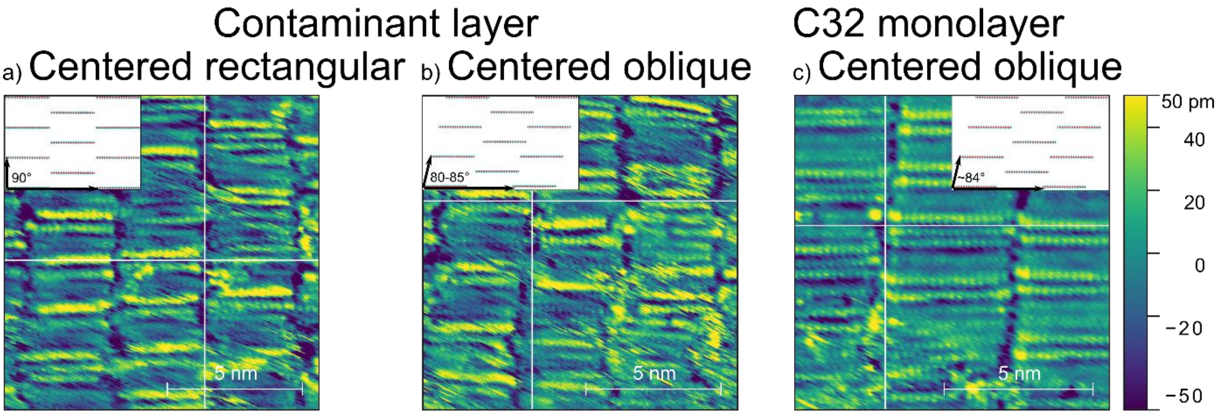

Supplementary Fig. 12. Low temperature (9K) STM images of the a-b) naturally contaminated and the c) vapor-deposited C32 monolayer. The structures of the regions are schematically drawn in the insets.

In Supplementary Fig. 12a-b we present low temperature STM images of the airborne contaminant layer which show two exemplary regions with two slightly different crystal structure. In Supplementary Fig. 12a the contaminant molecules self-organize into centered rectangular structure, as it can be seen schematically in the inset. In other regions, like in Supplementary Fig. 12b, we found that there is a minor deviation from this symmetric arrangement: the angle between the molecules' long axis and the lamellas are now between 80-85°.

As we discussed in the main text the airborne contaminant layer is composed of normal alkanes with 20-26 carbons atoms. The exact structure of the molecular monolayer of mid-length alkanes (16-21 carbon length) shows an odd/even alternation such that the even-length *n*-alkanes form oblique centered, while odd ones form rectangular centered lamella-molecular backbone structures[15]. The dependence on molecular-parity is still observable in longer alkanes, however there is only a 4-5° difference from 90° in the case of even alkanes. In agreement with the literature, we found centered oblique structure in the C32 monolayer with tilt angle of ~84°, as it can be seen in Supplementary Fig. 12c. The structure is schematically drawn in the inset.

Therefore, we conclude that the exact arrangements the contaminant alkane molecules show (in the above-mentioned minor) variations between regions thanks to their heterogenous lengths, the exact angle between the molecules and the lamellas may vary between 80-85° or 90°.

Supplementary Figure 13. – Control experiment with vapor deposited carboxylic acid molecular monolayer

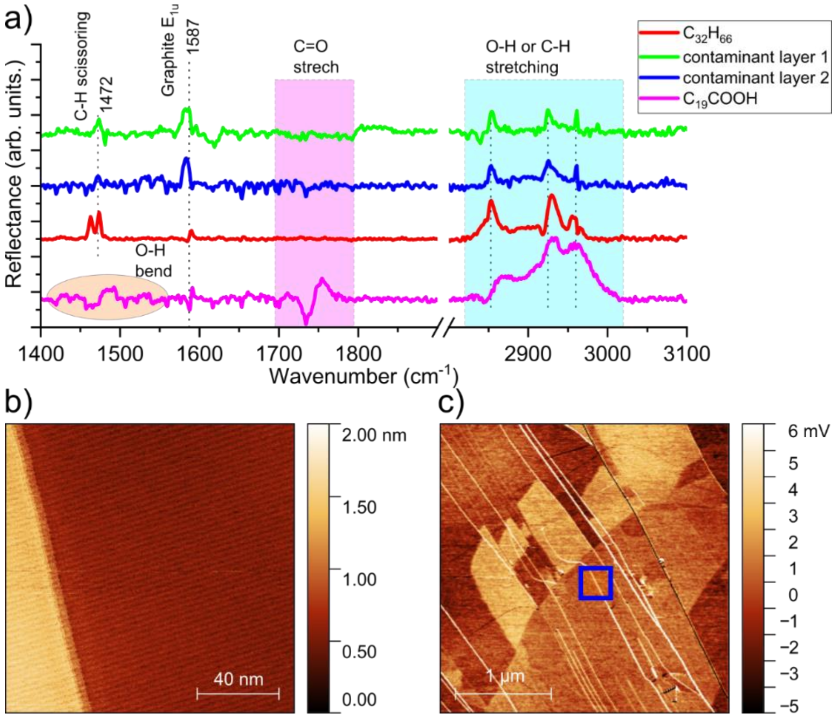

Supplementary Fig. 13. a) Grazing angle infrared spectra of differently covered graphite substrates, revealing the molecular vibrational modes of the adsorbates: airborne contaminant layer sample1 and sample2 green and blue respectively, dotriacontane ($C_{32}H_{66}$) red, arachidic acid ($C_{19}H_{39}COOH$) magenta. b) PF AFM Topography image reveals the stripe pattern of the self-organizing arachidic acid monolayer. Note that the stripes cross a step between terraces of graphite. c) Contact mode AFM torsion signal reveals frictional anisotropy domains on HOPG caused by the arachidic acid monolayer.

We deposited arachidic acid ($C_{19}H_{39}COOH$) monolayer from vapor phase onto HOPG, to verify that our equipment is truly capable of observing the IR-signal of possible functional groups. In the IR spectrum (magenta in Supplementary Fig. 13a) of the artificially deposited carboxylic acid monolayer we identified the characteristic bands of the COOH headgroup: C=O stretching and O-H bending modes between 1700 to 1800 cm^{-1} and between 1400 to 1500 cm^{-1}, respectively. Furthermore, the stretching modes between 2800 and 3000 cm⁻1 of the airborne contaminant and alkane (only C-H) monolayer are qualitatively different from those of the O-H consisting $C_{19}H_{39}COOH$. Within our limitations no specific functional groups (-OH, -COOH, -O-, -SH, etc.) can be observed in the IR spectrum of the contaminant layer, therefore we conclude that the contaminant consists of alkanes.

Mid-length carboxylic acids also self-organize on graphitic surfaces in a very similar way to alkanes, as they form lamellas (or stripes) and domains[16,17]. By PeakForce AFM we were able to image the stripes formed by the vapor deposited arachidic acid monolayer, see Supplementary Fig. 13b. We measured 2.68±0.2 nm for the period of the stripes/lamellas, in agreement with the all-trans length of 20 carbon length alkyl backbone. The self-organized arachidic acid monolayer also produced friction anisotropy domains thanks to its long alkyl backbone (see Supplementary Fig. 13c). We can conclude that besides the naturally occurring or artificially deposited alkane monolayers (see Figure 4 in the main text and Supplementary Fig. 7), other types of molecules which self-organize in similar fashion (eg. carboxyl acids or surfactants) may produce anisotropic frictional domains.

Supplementary references:

1. Khestanova, E., Guinea, F., Fumagalli, L., Geim, A. K. & Grigorieva, I. V. Universal shape and pressure inside bubbles appearing in van der Waals heterostructures. *Nat. Commun.* **7**, 12587 (2016).
2. Mao, J. X., Kroll, P. & Schug, K. A. Vacuum ultraviolet absorbance of alkanes: an experimental and theoretical investigation. *Struct. Chem.* **30**, 2217–2224 (2019).
3. Li, Z. *et al.* Effect of airborne contaminants on the wettability of supported graphene and graphite. *Nat. Mater.* **12**, 925–931 (2013).
4. Kozbial, A. *et al.* Understanding the intrinsic water wettability of graphite. *Carbon N. Y.* **74**, 218–225 (2014).
5. Kozbial, A. *et al.* Study on the surface energy of graphene by contact angle measurements. *Langmuir* **30**, (2014).
6. Andrew Kozbial. Understanding the Intrinsic Water Wettability of Graphite. (University of Pittsburgh, 2016).
7. Boinovich, L. B. *et al.* Origins of thermodynamically stable superhydrophobicity of boron nitride nanotubes coatings. *Langmuir* **28**, 1206–1216 (2012).
8. Kozbial, A., Gong, X., Liu, H. & Li, L. Understanding the Intrinsic Water Wettability of Molybdenum Disulfide (MoS 2). *Langmuir* **31**, 8429–8435 (2015).
9. Chow, P. K. *et al.* Wetting of mono and few-layered WS2 and MoS2 films supported on Si/SiO2 substrates. *ACS Nano* **9**, 3023–3031 (2015).
10. Choi, J. S. *et al.* Facile characterization of ripple domains on exfoliated graphene. *Rev. Sci. Instrum.* **83**, 073905 (2012).
11. Gallagher, P. *et al.* Switchable friction enabled by nanoscale self-assembly on graphene. *Nat. Commun.* **7**, 10745 (2016).
12. Paserba, K. R. & Gellman, A. J. Kinetics and Energetics of Oligomer Desorption from Surfaces. *Phys. Rev. Lett.* **86**, 4338–4341 (2001).
13. Paserba, K. R. & Gellman, A. J. Effects of conformational isomerism on the desorption kinetics of n-alkanes from graphite. *J. Chem. Phys.* **115**, 6737–6751 (2001).
14. Choi, J. S. *et al.* Friction anisotropy-driven domain imaging on exfoliated monolayer graphene. *Science* **333**, 607–610 (2011).
15. Espeau, P., Reynolds, P. A., Bowling, T., Cookson, D. & White, J. W. X-Ray diffraction from layers of n-alkanes adsorbed on graphite. *J. Chem. Soc. - Faraday Trans.* **93**, 3201–3208 (1997).
16. Rabe, J. P. & Buchholz, S. Commensurability and Mobility in Two-Dimensional Molecular Patterns on Graphite. *Science* **253**, 424–427 (1991).
17. Cyr, D. M., Venkataraman, B. & Flynn, G. W. STM Investigations of Organic Molecules Physisorbed at the Liquid−Solid Interface. *Chem. Mater.* **8**, 1600–1615 (1996).
